# Association Between the Aggregate Index of Systemic Inflammation (AISI) and Tirofiban Use During Primary Percutaneous Coronary Intervention in Patients with ST-Elevation Myocardial Infarction

**DOI:** 10.3390/medicina62010155

**Published:** 2026-01-13

**Authors:** Kemal Emrecan Parsova, Erkan Kahraman, Furkan Durak, Khagani Isgandarov, Yalcin Velibey

**Affiliations:** 1 Department of Cardiology, Koc University Hospital, Davutpasa Caddesi, No: 4, Topkapı Mahallesi, Istanbul 34010, Türkiye; 2 Department of Cardiology, Siyami Ersek Thoracic and Cardiovascular Surgery Center, Training and Research Hospital, Istanbul 34668, Türkiyedrfurkandurak@gmail.com (F.D.);; 3 Department of Cardiology, Kartal Kosuyolu, Training and Research Hospital, Istanbul 34865, Türkiye

**Keywords:** inflammation, AISI, GP IIb/IIIa inhibitor, tirofiban, STEMI, primary PCI

## Abstract

*Background and Objectives*: Inflammation contributes to plaque rupture and thrombosis in ST-elevation myocardial infarction (STEMI). The Aggregate Index of Systemic Inflammation (AISI) is a novel biomarker that reflects innate immune and thrombotic activation. Due to the connection between inflammation and thrombosis, higher AISI values could indicate a greater thrombus burden and the necessity of glycoprotein IIb/IIIa inhibitors. The aim of this study was to assess the relationship between AISI and tirofiban use during primary percutaneous coronary intervention (PCI) in STEMI patients. *Materials and Methods*: This retrospective study included 2624 STEMI patients who underwent primary PCI at a tertiary heart center between 2019 and 2024. Patients with pre-hospital fibrinolysis, missing laboratory data, or rescue PCI were excluded. AISI was calculated as (neutrophil × monocyte × platelet)/lymphocyte. The primary outcome was tirofiban use during PCI. Univariate and multivariable logistic regression analyses were performed to identify independent predictors, and receiver operating characteristic (ROC) curve analysis was used to evaluate AISI performance. Statistical significance was defined as *p* < 0.05. *Results*: Among the 2624 patients with STEMI undergoing primary PCI, tirofiban was administered in 23.5% of cases. Patients receiving tirofiban had significantly higher AISI values (*p* < 0.001). ROC analysis demonstrated that AISI predicted tirofiban use with a modest discriminative performance (AUC = 0.566; 95% CI 0.536–0.596; *p* < 0.001). In multivariable logistic regression, younger age (OR 0.98; *p* < 0.001), higher AISI (per 100-unit increase; OR 1.01; *p* = 0.037), and lower LVEF (OR 0.98; *p* < 0.001) independently predicted tirofiban use, whereas admission glucose showed only borderline significance (*p* = 0.089). *Conclusions*: Elevated AISI was independently associated with tirofiban use during primary PCI, indicating that systemic inflammatory status parallels intraprocedural decision-making in STEMI. Although its discriminative performance was modest, AISI reflects systemic inflammatory–thrombotic activation in this clinical setting.

## 1. Introduction

Cardiovascular disease (CVD) remains the leading cause of death and morbidity worldwide [1]. ST-elevation myocardial infarction (STEMI) typically results from the disruption, rupture, or erosion of an unstable atherosclerotic plaque in a coronary artery, leading to complete vessel occlusion, transmural myocardial ischemia, and infarction [2,3]. Recent advances in vascular biology have transformed our understanding of atherosclerosis, shifting the perspective from viewing it as merely a lipid-storage disease to viewing it as a chronic, immune-mediated inflammatory process [4,5]. The initiation of atherosclerosis involves endothelial dysfunction, increased expression of adhesion molecules, and migration of inflammatory cells into the subendothelial space, leading to foam cell formation and plaque development [5,6]. Not only does inflammation play a central role in the initiation and progression of atherosclerotic lesions, but it also plays a role in their acute complications, which lead to plaque rupture/erosion and thrombosis [4,5,6]. In the context of acute coronary syndrome (ACS), elevated levels of inflammatory markers, such as C-reactive protein (CRP), interleukin (IL)-6, and serum amyloid A, have been shown to correlate with negative outcomes [7,8].

Both experimental and clinical studies demonstrate that inflammatory processes promote thrombus formation through multiple mechanisms, including endothelial activation, enhanced platelet aggregation, and inhibition of endogenous fibrinolysis [6,8,9]. Activated neutrophils and monocytes release proteolytic enzymes and procoagulant mediators, and platelets further amplify the inflammatory response by releasing cytokines and recruiting leukocytes [7,8]. Consequently, inflammation and thrombosis act as interdependent, self-reinforcing processes in ACS pathogenesis. Inflammation-induced hypercoagulability can increase thrombus burden in the infarct-related artery, complicating primary percutaneous coronary intervention (PCI) and influencing periprocedural management strategies [9].

The Aggregate Index of Systemic Inflammation (AISI) has recently emerged as a novel biomarker that integrates multiple components of both innate immunity and thrombocytic activation [10,11]. Recent studies have demonstrated that elevated AISI values are associated with the presence and severity of coronary artery disease and with adverse outcomes in patients with ACS, particularly in those undergoing PCI [10,11,12,13].

Glycoprotein IIb/IIIa inhibitors, such as tirofiban, play a crucial role in the management of thrombotic complications during PCI. By reversibly blocking the final common pathway of platelet aggregation, tirofiban effectively prevents fibrinogen-mediated platelet cross-linking [14]. In patients with STEMI, its use has been shown to reduce thrombus burden, improve microvascular perfusion, and enhance post-procedural TIMI flow, particularly in cases with large intracoronary thrombus or no-reflow phenomenon. However, due to the associated bleeding risk, current guidelines recommend selective administration in patients with high thrombotic load or inadequate flow restoration despite optimal antiplatelet therapy [9,15].

By capturing the cumulative effect of neutrophil, monocyte, and platelet activation relative to lymphocyte suppression, the AISI may represent a more accurate indicator of inflammation-driven thrombosis, which could influence intraprocedural pharmacologic strategies such as the use of glycoprotein IIb/IIIa inhibitors like tirofiban.

Despite the biological plausibility linking systemic inflammation to thrombotic burden, the association between AISI and intraprocedural glycoprotein IIb/IIIa inhibitor use in STEMI has not been clearly defined. Therefore, this study aimed to investigate whether the AISI could serve as an independent predictor of tirofiban use during primary PCI in patients presenting with STEMI.

## 2. Materials and Methods

### Patient Selection

This retrospective, single-center study included 2624 patients diagnosed with STEMI who underwent primary PCI at a tertiary heart center between January 2019 and December 2024. All patients were admitted through the regional STEMI network and underwent urgent coronary angiography within 12 h of symptom onset. Patients who had received pre-hospital fibrinolytic therapy, had missing hematologic or biochemical data, underwent rescue PCI, or were managed conservatively were excluded. After exclusions, the final study population consisted of 2624 patients.

All patients received a loading dose of acetylsalicylic acid (300 mg) and one of the following P2Y_12_ inhibitors before PCI: clopidogrel (600 mg), prasugrel (60 mg), or ticagrelor (180 mg). In addition, intravenous unfractionated heparin (70–100 U/kg) was administered prior to coronary instrumentation. The use of glycoprotein IIb/IIIa inhibitors (tirofiban; 25 µg/kg bolus followed by 0.15 µg/kg/min infusion for up to 18–24 h) was left to the operator’s discretion, based on the angiographic thrombus burden, no-reflow, or high thrombotic risk, in accordance with institutional protocol and guideline recommendations [15,16]. Accordingly, tirofiban administration in this study reflects a real-world, operator-driven procedural decision integrating angiographic thrombus appearance, procedural flow characteristics, ischemic time, hemodynamic status, and perceived thrombotic risk, rather than a direct or standardized measurement of intracoronary thrombus burden. At our high-volume tertiary STEMI center, tirofiban administration was guided by guideline recommendations and was frequently initiated in patients presenting with high thrombus burden, delayed symptom-to-balloon time, or angiographic features suggestive of impending slow-flow/no-reflow. During the study period, institutional practice favored relatively liberal use of glycoprotein IIb/IIIa inhibitors in high-risk cases. Consequently, the rate of tirofiban use in our cohort reflects both therapeutic and preventive administration based on real-time operator assessment of thrombotic risk, rather than the incidence of angiographically confirmed no-reflow. All PCI procedures were performed using standard techniques with drug-eluting stents. The culprit lesion was identified based on the patient’s clinical presentation, ECG findings, and angiographic characteristics.

The study was conducted in accordance with the Declaration of Helsinki (1975) and its subsequent revisions. All patients provided informed consent prior to inclusion, and ethical approval was obtained from the institutional ethics committee (decision number E-10840098-202.3.02-5847, Date: 31 August 2025).

## 3. Laboratory Analysis

Blood samples were obtained upon admission for a complete blood count and routine biochemistry. The estimated glomerular filtration rate (eGFR) was calculated using the CKD-EPI formula. Patients with eGFR < 30 mL/min/1.73 m^2^ were classified as having severe chronic kidney disease (CKD). Anemia was defined as hemoglobin (HGB) < 12 g/dL on admission. AISI was calculated using the following formula: AISI = (neutrophil × monocyte × platelet)/lymphocyte. Patients were divided into two groups based on the median value of AISI: high AISI and low AISI groups. Left ventricular ejection fraction (LVEF) was assessed within the first 24 h after admission using transthoracic echocardiography.

## 4. Definitions

The diagnosis of STEMI was established according to the international guidelines [15,16]. Patients were considered to have STEMI if they presented with ischemic symptoms lasting more than 30 min and persistent ST-segment elevation or an ST-elevation equivalent pattern on electrocardiogram (ECG) consistent with acute coronary artery occlusion. ST-segment elevation was defined as new elevation at the J-point in at least two contiguous leads meeting any of the following criteria: ≥2.5 mm in men <40 years, ≥2.0 mm in men ≥40 years, or ≥1.5 mm in women regardless of age in leads V2–V3, and/or ≥1.0 mm in other leads, in the absence of left ventricular hypertrophy or left bundle branch block. Patients with left bundle branch block, right bundle branch block, or paced rhythm and a high clinical suspicion of acute ischemia were also managed as STEMI equivalents [15,16].

The culprit lesion was identified based on the patient’s clinical presentation, ECG findings, and angiographic characteristics. Successful PCI was defined as achieving final Thrombolysis in Myocardial Infarction (TIMI) grade 3 flow in the infarct-related artery, with residual stenosis < 20% (or <30% following balloon angioplasty) after stent implantation, and no need for emergency coronary artery bypass grafting (CABG). Unsuccessful PCI was defined as final TIMI flow ≤ 2, residual stenosis ≥ 30%, the need for emergency CABG, or intra-procedural death.

## 5. Statistical Analysis

All statistical analyses were conducted using IBM SPSS Statistics version 25.0 (IBM Corp., Armonk, NY, USA). Categorical variables are reported as counts and percentages. Continuous variables are presented as mean ± standard deviation for normally distributed data and as median with interquartile range for non-normally distributed data. Data normality was evaluated using the Kolmogorov–Smirnov or Shapiro–Wilk tests, as appropriate. Comparisons between groups were performed using the chi-square or Fisher’s exact test for categorical variables, the independent samples *t*-test for normally distributed continuous variables, and the Mann–Whitney U test for non-normally distributed continuous variables. To identify independent predictors of tirofiban use during primary PCI, we constructed a multivariable logistic regression model with covariates selected a priori based on clinical relevance and biological plausibility, rather than on univariate statistical significance. All clinically relevant covariates were retained in the multivariable model irrespective of their univariate associations. Results are reported as odds ratios (OR) with 95% confidence intervals (CI). The ability of AISI to discriminate patients who received tirofiban was assessed using receiver operating characteristic (ROC) curve analysis, with the area under the curve (AUC) used as a measure of discriminative performance. The median AISI value was used for primary group-based comparisons, whereas the ROC analysis served as a secondary exploratory assessment to evaluate the discriminative ability of AISI for predicting tirofiban use. The ROC-derived cutoff was not used for stratification. A *p*-value of <0.05 was considered statistically significant.

## 6. Results

A total of 2624 patients (mean age = 61.8 ± 12.1 years; 78% male) presenting with STEMI and undergoing primary PCI were included in the analysis. Table 1 summarizes the baseline demographic, clinical, and laboratory characteristics of the study population. The median AISI value of the entire cohort was 697.5 (IQR: 394.1–1219.8). Patients were stratified into two groups based on the median AISI value (AISI = 697.5): those with AISI ≤ 697.5 (low-AISI group, n = 1312) and AISI > 697.5 (high-AISI group, n = 1312). Patients in the high-AISI group exhibited a more adverse clinical and biochemical profile. They demonstrated significantly higher admission glucose, creatinine, and blood urea nitrogen (BUN) levels, together with a markedly lower LVEF. Severe CKD was also substantially more common among patients with elevated AISI values (8.2% vs. 3.4%, *p* < 0.001). Furthermore, anterior MI occurred with greater frequency in the high-AISI group (54.9% vs. 41.8%, *p* < 0.001). Procedural outcomes similarly differed between groups: unsuccessful PCI was significantly more prevalent in the high-AISI cohort (7.4% vs. 4.0%, *p* < 0.001), and the use of tirofiban during primary PCI was notably higher among patients with elevated AISI values (14.7% vs. 32.1%, *p* < 0.001) (Table 2).

In univariate logistic regression analyses, younger age, higher admission glucose, lower LVEF, and higher AISI were associated with tirofiban use. Severe CKD and anterior myocardial infarction were not significant in univariate analyses. In the multivariable logistic regression model (specified a priori using clinically relevant covariates), younger age (OR: 0.98; 95% CI: 0.97–0.99; *p* < 0.001), higher AISI (per 100-unit increase; OR: 1.01; 95% CI: 1.00–1.01; *p* = 0.037), and lower LVEF (OR: 0.98; 95% CI: 0.97–0.99; *p* < 0.001) remained independently associated with tirofiban use. Admission glucose showed a borderline association (*p* = 0.089), whereas severe CKD and anterior myocardial infarction were not independently associated with tirofiban use (Table 3).

**Figure 1:**
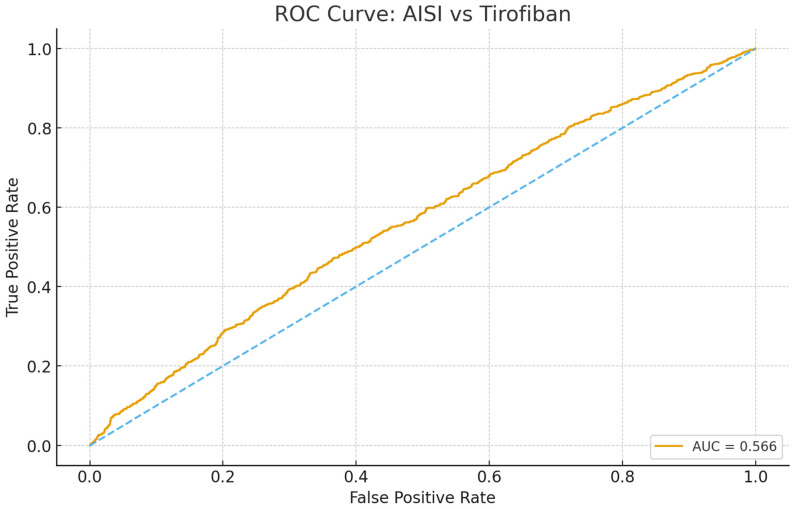
ROC curve of AISI for predicting tirofiban use during primary PCI.

ROC curve analysis demonstrated that AISI exhibited a modest but statistically significant discriminatory ability for predicting the need for tirofiban during primary PCI (AUC = 0.566, 95% CI: 0.536–0.596; *p* < 0.001), as illustrated in Figure 1.

Taken together, these findings indicate that higher AISI values are associated with procedural characteristics prompting tirofiban administration, lower procedural success (TIMI ≤ 2), and reduced left ventricular systolic function, underscoring the interplay between systemic inflammation and adverse procedural characteristics in STEMI.

## 7. Discussion

In this study, higher AISI values were significantly associated with the use of tirofiban during primary PCI in patients with STEMI. Although the discriminative performance of AISI was modest, it remained independently associated with tirofiban administration after adjustment for key clinical factors. These findings indicate that AISI, as an integrative marker of systemic inflammation and thrombocytosis, reflects a heightened inflammatory–thrombotic milieu that parallels intraprocedural antiplatelet treatment decisions in real-world practice [10,17].

AISI is calculated as (neutrophils × monocytes × platelets)/lymphocytes, thereby combining three cellular mediators of innate immune and thrombotic activity against the counter-regulatory adaptive lymphocyte response. Neutrophils and monocytes are early responders to plaque rupture and actively promote thrombus formation by releasing proteolytic enzymes, tissue factor, and proinflammatory cytokines [8,18]. Platelets interact with leukocytes via P-selectin and CD40L pathways, leading to endothelial activation and propagation of the inflammatory cascade. Lymphopenia, conversely, is a hallmark of stress-induced immunosuppression and is associated with adverse cardiovascular outcomes [19,20,21]. Thus, a high AISI simultaneously captures neutrophil and monocyte activation, platelet hyperreactivity, and lymphocytic depletion—reflecting a state of systemic “immune-thrombotic imbalance.”

Previous studies have validated the prognostic relevance of AISI in various cardiovascular and systemic inflammatory conditions. Liu et al. (2025) showed that elevated AISI values independently predicted long-term mortality in patients with coronary artery disease undergoing PCI [10]. Hou et al. (2024) reported that AISI was among the strongest predictors of in-stent restenosis using machine learning models [17]. Similarly, Tuzimek et al. (2024) found significant correlations between AISI and acute coronary syndrome ACS incidence, particularly in diabetic and prediabetic populations [13].

Mechanistically, systemic inflammation enhances platelet aggregation and fibrin formation, promoting thrombus persistence and distal embolization [14]. Activated neutrophils release neutrophil extracellular traps, which provide a scaffold for platelet adhesion and thrombus stabilization. Elevated AISI may therefore signify extensive leukocyte–platelet cross-talk and a higher intracoronary thrombus burden. This could explain why patients with elevated AISI values in our study were more likely to require glycoprotein IIb/IIIa inhibitors such as tirofiban. Indeed, recent data have linked inflammatory cell activation to impaired microvascular reperfusion and no-reflow phenomena following primary PCI [22].

Our study demonstrated that both higher AISI values and reduced left ventricular ejection fraction were independently associated with the use of tirofiban during primary PCI, suggesting that systemic inflammatory activation and impaired myocardial function contribute to the procedural decision-making process. Reduced LVEF likely reflects a larger ischemic burden and a higher propensity for microvascular dysfunction, circumstances in which adjunctive glycoprotein IIb/IIIa inhibition is more frequently employed. Although anterior myocardial infarction was more common among patients with elevated AISI values, it did not remain an independent predictor after multivariable adjustment, indicating that infarct location alone may be insufficient to account for the need for intensified antiplatelet therapy.

The ability of AISI to discriminate patients who required tirofiban was modest, as reflected by an AUC of 0.566, underscoring that AISI is not a standalone determinant of procedural strategy. Importantly, intraprocedural tirofiban use should not be interpreted as a direct surrogate of angiographically quantified thrombus burden or microvascular failure. Rather, it represents a composite procedural endpoint reflecting operator-assessed thrombotic risk under dynamic clinical and angiographic conditions. In this context, our findings indicate that higher systemic inflammatory activity, as captured by AISI, parallels the likelihood of intensified antiplatelet therapy during primary PCI in real-world practice, without implying causality or objective thrombus measurement. Nevertheless, this modest discriminatory power does not negate its mechanistic relevance. AISI integrates leukocyte activation, thrombocytic reactivity, and lymphocytic suppression—components known to drive intracoronary thrombus formation and distal embolization. Thus, while its predictive performance is limited at the individual level, AISI still captures a biologically meaningful inflammatory–thrombotic profile that provides complementary biological insight into procedural risk in STEMI. Future studies integrating AISI with angiographic thrombus grading, microvascular perfusion indices, or imaging-based assessments may help clarify whether combined models can enhance prediction of adjunctive pharmacologic needs during primary PCI.

## 8. Study Limitations

Nevertheless, this study has several limitations. This is a retrospective, single-center study. Tirofiban administration depended on the discretion of the operator, which may vary with angiographic interpretation. Therefore, the present analysis should be viewed as exploring the relationship between systemic inflammatory status and procedural decision-making, rather than direct pathophysiological assessment of thrombus burden. Furthermore, AISI was only assessed upon admission. Serial measurements might provide more dynamic insights into the evolution of inflammation and thrombus burden. Future multicenter, prospective studies are needed to validate these findings and investigate whether AISI-guided therapeutic strategies can optimize outcomes and reduce ischemic complications in STEMI.

## 9. Conclusions

In patients presenting with STEMI and undergoing primary PCI, elevated AISI levels were independently associated with the use of tirofiban, suggesting that heightened systemic inflammation parallels intraprocedural decision-making in real-world practice. Although its discriminative performance was modest, AISI reflects a biologically meaningful inflammatory–thrombotic milieu rather than a standalone clinical decision tool. Future prospective multicenter studies incorporating detailed procedural and angiographic variables are warranted to further define its potential role in integrated risk assessment.

## Figures and Tables

**Table 1:** Baseline demographic and clinical characteristics of the study population.

Variable	Overall (n = 2624)
Age (years, mean ± SD)	61.8 ± 12.1
Male sex, n (%)	2045 (78.0)
Hypertension, n (%)	1343 (51.2)
Diabetes mellitus, n (%)	725 (27.6)
Current smoking, n (%)	1116 (42.5)
Hyperlipidemia, n (%)	968 (36.9)
Severe chronic kidney disease (eGFR < 30 mL/min/1.73 m^2^), n (%)	152 (5.8)
Anemia (Hgb < 12 g/dL), n (%)	376 (14.3)
Prior PCI, n (%)	301 (11.5)
Prior CABG, n (%)	142 (5.4)
COPD, n (%)	171 (6.5)
Stroke history, n (%)	94 (3.6)
Admission glucose (mg/dL)	142.4 ± 47.6
Creatinine (mg/dL)	1.08 ± 0.31
BUN (mg/dL)	18.7 ± 7.5
eGFR (mL/min/1.73 m^2^)	82.5 ± 18.4
Hemoglobin (g/dL)	13.7 ± 1.8
Platelet count (×10^3^/µL)	236 ± 68
Neutrophil (×10^3^/µL)	7.8 ± 2.6
Lymphocyte (×10^3^/µL)	1.9 ± 0.7
Monocyte (×10^3^/µL)	0.65 ± 0.23
AISI	850 ± 340
LVEF (%)	47.5 ± 9.9
Successful PCI (TIMI 3) n (%)	2457 (93.6)
Unsuccessful PCI (TIMI ≤ 2) n (%)	167 (6.4)
Tirofiban use n (%)	617 (23.5)

Abbreviations: eGFR—estimated glomerular filtration rate; PCI—percutaneous coronary intervention; CABG—coronary artery bypass grafting; COPD—chronic obstructive pulmonary disease; BUN—blood urea nitrogen; AISI—aggregate index of systemic inflammation; LVEF—left ventricular ejection fraction; TIMI—thrombolysis in myocardial infarction.

**Table 2:** Comparison of clinical and laboratory characteristics according to AISI median groups.

Variable	Low AISI (≤697.5) (n = 1312)	High AISI (>697.5) (n = 1312)	*p*-Value
Age (years)	59.2 ± 11.8	64.4 ± 11.9	<0.001
Male sex, n (%)	1001 (76.3)	1044 (79.6)	0.041
Hypertension, n (%)	601 (45.8)	742 (56.6)	<0.001
Diabetes mellitus, n (%)	301 (22.9)	424 (32.3)	<0.001
Severe chronic kidney disease, n (%)	45 (3.4)	107 (8.2)	<0.001
Admission glucose (mg/dL)	132.5 ± 41.2	152.3 ± 50.1	<0.001
Creatinine (mg/dL)	1.04 ± 0.27	1.13 ± 0.34	<0.001
BUN (mg/dL)	17.4 ± 6.8	20.1 ± 8.0	<0.001
eGFR (mL/min/1.73 m^2^)	84.8 ± 16.9	80.3 ± 19.6	<0.001
LVEF (%)	49.3 ± 9.2	45.6 ± 10.3	<0.001
Anterior MI, n (%)	548 (41.8)	721 (54.9)	<0.001
Successful PCI, n (%)	1267 (96.6)	1210 (92.1)	0.002
Unsuccessful PCI, n (%)	45 (3.4)	102 (7.9)	0.002
Tirofiban use, n (%)	193 (14.7)	424 (32.1)	<0.001

Abbreviations: BUN—blood urea nitrogen; eGFR—estimated glomerular filtration rate; LVEF—left ventricular ejection fraction; MI—myocardial infarction; PCI—percutaneous coronary intervention.

**Table 3:** Univariate and multivariable logistic regression analyses for predictors of tirofiban use. (Multivariable model covariates were selected a priori based on clinical relevance and were retained irrespective of univariate associations.).

Variable	Univariate OR (95% CI)	*p*	Multivariable OR (95% CI)	*p*
Age (per 1 year)	0.98 (0.98–0.99)	<0.001	0.98 (0.97–0.99)	<0.001
Severe CKD	0.58 (0.24–1.38)	0.217	0.59 (0.21–1.65)	0.31
Anterior MI	1.15 (0.99–1.35)	0.066	0.99 (0.83–1.18)	0.91
AISI (per 100-unit increase)	1.01 (1.01–1.02)	<0.001	1.01 (1.00–1.01)	0.037
LVEF (%)	0.98 (0.98–0.99)	<0.001	0.98 (0.97–0.99)	<0.001
Admission glucose (mg/dL)	1.001 (1.000–1.002)	0.044	1.001 (1.000–1.002)	0.089

Abbreviations: CKD—chronic kidney disease; MI—myocardial infarction; AISI—aggregate index of systemic inflammation; LVEF—left ventricular ejection fraction.

## Data Availability

Data are available from the corresponding author upon reasonable request.
